# Sleep disturbances in people with depression: A semi-structured interview study of treatment methods and patients’ experiences within psychiatric practice

**DOI:** 10.1192/j.eurpsy.2026.12093

**Published:** 2026-07-31

**Authors:** C. Moesgaard, P. Hjorth

**Affiliations:** 1 Psychiatric, Region of Southern Denmark, Vejle; 2 Institute of Regional Health Research, Region of Southern Denmark, Odense, Denmark

## Abstract

**Introduction:**

Major depressive disorder (MDD) is characterized by profound sadness, low energy, and fatigue. Sleep disturbances affect 75–90% of patients and increase suicide risk (WHO 2017). Sleep is essential for brain, immune, metabolic, and cardiovascular health (Vorster et al. Clin Transl Neurosci 2024; 8:8). Despite guidelines, sleep problems remain common in psychiatric populations (Nutt et al. Dialogues Clin Neurosci 2008; 10:329–36). This study explores treatment and counseling practices for depression-related sleep disturbances to inform outpatient care.

**Objectives:**

To examine how patients with MDD experience current treatments for sleep disturbances and to identify ways to strengthen clinical practice with pharmacological and non-pharmacological approaches.

**Methods:**

Using a hermeneutic, inductive design, semi-structured interviews were conducted with 12 patients diagnosed with MDD and reporting sleep disturbances. Participants were recruited through psychiatric outpatient clinics in Denmark via providers, conferences, emails, and flyers. Interviews (45–50 minutes) were carried out between February and April 2024 in clinics or patients’ homes. The study followed SRQR guidelines (EQUATOR Network 2023), adhered to the Helsinki Declaration (World Medical Association 2008), and was approved by the Danish ethics committee (protocol no. 20232000-151).

**Results:**

Analysis is ongoing, applying Braun and Clarke’s thematic content analysis (Braun & Clarke. Qual Res Psychol 2006; 3:75–101) with NVivo software (Zamawe. Malawi Med J 2015; 27:13–5). Preliminary findings indicate a predominant reliance on pharmacological treatments, often linked to adverse effects, while awareness of non-pharmacological options appears limited. Early interviews highlight a need for enhanced training among healthcare professionals to broaden treatment strategies.

**Conclusions:**

This study will provide insights into patients’ perspectives on managing sleep disturbances in MDD. Early results suggest over-reliance on pharmacological therapy and limited use of alternatives. Final conclusions and clinical implications will be presented at the conference.

**Disclosure of Interest:**

None Declared

